# Experimental and Computational Approaches for Solubility Measurement of Pyridazinone Derivative in Binary (DMSO + Water) Systems

**DOI:** 10.3390/molecules25010171

**Published:** 2019-12-31

**Authors:** Faiyaz Shakeel, Sultan Alshehri, Mohd Imran, Nazrul Haq, Abdullah Alanazi, Md. Khalid Anwer

**Affiliations:** 1 Department of Pharmaceutics, College of Pharmacy, King Saud University, P.O. Box 2457, Riyadh 11451, Saudi Arabia; salshehri1@ksu.edu.sa (S.A.); nazrulhaq59@gmail.com (N.H.); alanazylab@gmail.com (A.A.); 2 Department of Pharmaceutical Chemistry, Faculty of Pharmacy, Northern Border University, P.O. Box 840, Rafha 91911, Saudi Arabia; imran_inderlok@yahoo.co.in; 3 Department of Pharmaceutics, College of Pharmacy, Prince Sattam bin Abdulaziz University, P.O. Box 173, Al-Kharj 11942, Saudi Arabia; mkanwer2002@yahoo.co.in

**Keywords:** computational models, pyridazinone derivative, Solution thermodynamics, solubilization

## Abstract

The current research work was performed to evaluate the solubilization behavior, solution thermodynamics, and solvation behavior of poorly soluble pyridazinone derivative i.e., 6-phenyl-pyridazin-3(2*H*)-one (PPD) in various binary solvent systems of dimethyl sulfoxide (DMSO) and water using experimental and various computational approaches. The solubility of PPD in various binary solvent system of DMSO and water was investigated within the temperature range *T* = 298.2 K to 318.2 K at constant air pressure *p* = 0.1 MPa, by employing an isothermal technique. The generated solubility data of PPD was computationally represented by five different cosolvency models including van’t Hoff, Apelblat, Yalkowsky–Roseman, Jouyban–Acree, and Jouyban–Acree–van’t Hoff models. The performance of each computational model for correlation studies was illustrated using root mean square deviations (*RMSD*). The overall *RMSD* value was obtained <2.0% for each computational model. The maximum solubility of PPD in mole fraction was recorded in neat DMSO (4.67 × 10^−1^ at *T* = 318.2 K), whereas the lowest one was obtained in neat water (5.82 × 10^−6^ at *T* = 298.2 K). The experimental solubility of PPD in mole fraction in neat DMSO was much higher than its ideal solubility, indicating the potential of DMSO for solubility enhancement of PPD. The computed values of activity coefficients showed maximum molecular interaction in PPD-DMSO compared with PPD-water. Thermodynamic evaluation showed an endothermic and entropy-driven dissolution of PPD in all the mixtures of DMSO and water. Additionally, enthalpy–entropy compensation evaluation indicated an enthalpy-driven mechanism as a driven mechanism for the solvation property of PPD.

## 1. Introduction

**Figure 1:**
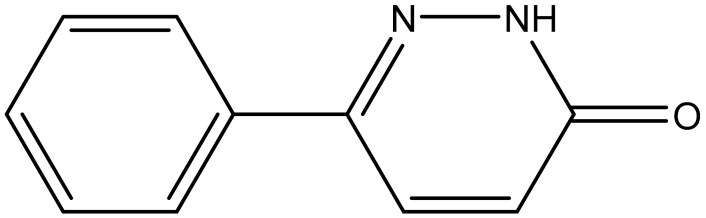
Chemical structure of PPD.

The investigated molecule 6-phenylpyridazin-3(2*H*)-one (PPD) [chemical structure: Figure 1; chemical name: 6-phenylpyridazin-3(2*H*)-one; molecular formula: C_10_H_8_N_2_O; molecular weight: 172.18 g mol^−1^ and CASRN: 2166-31-6] is a pyridazinone derivative which is being used as cardiotonic agent [1,2].

Some other biological activities, including insecticidal [3], cardioprotective [4,5], analgesics [6,7], anti-inflammatory [7,8], antinociceptive [1], antiulcer [9], and antimicrobial activity [10], have also been reported for different PPD derivatives. The main problem of PPD and related compounds is high toxicity and week solubilization potential in an aqueous media [1,11]. Hence, the solubilities and other physicochemical information about these molecules in aqueous-cosolvent binary systems are important for their complete physicochemical characterization [12,13]. The potential of dimethyl sulfoxide (DMSO) in enhancing the solubility of various weakly soluble drugs such as 6-methyl-2-thiouracil, sinapic acid, naringin and bergenin have been proved in the literature [12,14,15,16]. The solubility data and other physicochemical properties of PPD have been reported poorly in the literature. The solubility of PPD in neat DMSO and neat water has been reported recently [17]. The solubility and solution thermodynamic properties of PPD in various DMSO + water systems are not reported elsewhere. Hence, in the proposed study, the solubility, solution thermodynamic properties and solvation behavior of PPD in binary DMSO + water systems and neat solvents were studied using experimental and various computational approaches. The solubility of PPD was determined by applying an isothermal technique within temperature range *T* = 298.2 K to 318.2 K at constant air pressure *p* = 0.1 MPa. The impact of pressure on the solubility of PPD was not studied in this work and hence these studies were carried out at constant air pressure i.e., *p* = 0.1 MPa. The temperature range *T* = 298.2 K to 318.2 K was maintained in this range in such a way that the maximum investigated temperature (i.e., *T* = 318.2 K) should not exceeds the melting temperature of PPD and boiling points of the studied solvents. The melting temperature of PPD has been found as 476.43 K in our previous work [17]. The boiling points of water and DMSO are 373.2 and 462.2 K, respectively. The maximum investigated temperature (i.e., *T* = 318.2 K) was found much lower than melting temperature of PPD and boiling points of water and DMSO and hence the proposed temperature range was maintained in this study. Activity coefficients of PPD and various cosolvent mixtures were computed using ideal and experimental solubility data of PPD. Using activity coefficients, solute-solvent interactions at molecular level were evaluated. The physicochemical and solubility data of PPD obtained in this work would motivate the pharmaceutical scientists to obtain similar data for newly synthesized medicinal compounds as well as for already existing compounds [18,19,20]. The solubility data generated in the proposed study would be helpful in purification, recrystallization, drug discovery process, and dosage form design of PPD.

## 2. Results and Discussion

### 2.1. Solubility of PPD in Binary DMSO + Water Systems

The experimental mole fraction solubility (*x*_e_) values of PPD in binary DMSO + water systems and neat solvents at *T* = 298.2 K to 318.2 K and *p* = 0.1 MPa are tabulated in Table 1.

**Figure 2:**
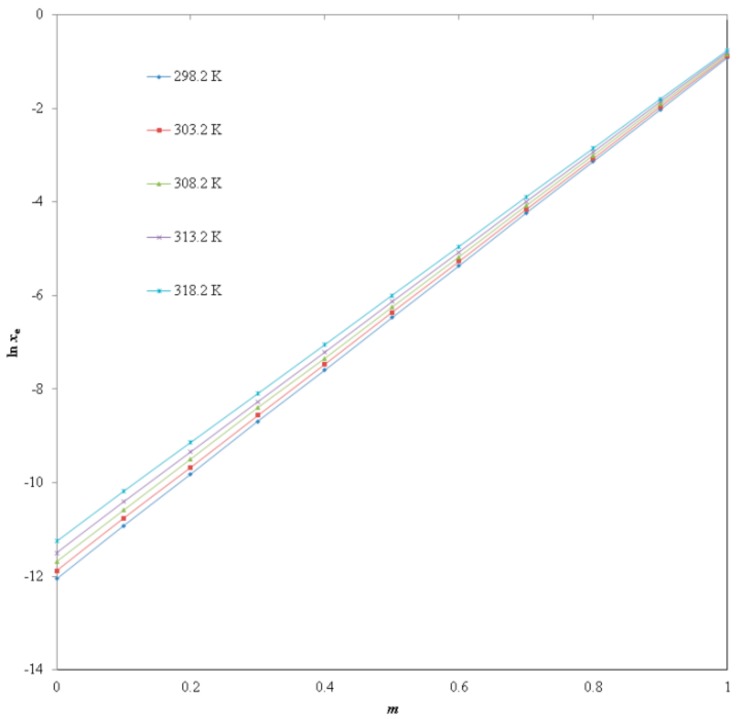
Impact of *m* value of the DMSO on ln *x*_e_ values of PPD at five different temperatures i.e., *T* = 29.2 K to 318.2 K.

The solubility of PPD in neat DMSO and neat water has been reported at *T* = 298.2 K to 318.2 K and *p* = 0.1 MPa [17]. The solubility of PPD in mole fraction in pure DMSO and pure water at *T* = 298.2 K was estimated as 4.03 × 10^−1^ (*m* = 1.0) and 5.75 × 10^−6^ (*m* = 0.0), respectively in the literature [17]. The solubility of PPD in mole fraction in pure DMSO and pure water at *T* = 298.2 K was recorded as 4.00 × 10^−1^ (*m* = 1.0) and 5.82 × 10^−6^ (*m* = 0.0), respectively in the present work. The solubility of PPD in mole fraction in water (*m* = 0.0) at other temperatures i.e., *T* = 303.2, 308.2, 313.2 and 318.2 K was found as 6.91 × 10^−6^, 8.37 × 10^−6^, 1.00 × 10^−5^ and 1.26 × 10^−5^, respectively in the literature [17]. The solubility of PPD in mole fraction in water (*m* = 0.0) at *T* = 303.2, 308.2, 313.2 and 318.2 K was recorded 6.94 × 10^−6^, 8.42 × 10^−6^, 1.02 × 10^−5^ and 1.30 × 10^−5^, respectively in the present study. The solubility of PPD in mole fraction in DMSO (*m* = 1.0) at *T* = 303.2, 308.2, 313.2 and 318.2 K was found as 4.19 × 10^−1^, 4.38 × 10^−1^, 4.55 × 10^−1^ and 4.73 × 10^−1^, respectively in the literature [17]. The solubility of PPD in mole fraction in DMSO (*m* = 1.0) at *T* = 303.2, 308.2, 313.2 and 318.2 K was recorded as 4.16 × 10^−1^, 4.32 × 10^−1^, 4.49 × 10^−1^ and 4.67 × 10^−1^, respectively in the present study. Overall, the recorded solubilities of PPD in DMSO and water at *T* = 298.2 K to 318.2 K were very close to the literature values. The solubility of PPD was found to increase linearly with raise in temperature and in all cases the solubility of PPD increases as the proportion of DMSO in binary DMSO + water system increases. The highest solubility of PPD in mole fraction was obtained in neat DMSO (4.67 × 10^−1^ at *T* = 318.2 K), whereas, the lowest one was found in neat water (5.82 × 10^−6^ at *T* = 298.2 K). The highest solubility of PPD in neat DMSO was possible due to lower polarity of DMSO as compared with water [12,16]. The effect of mass fraction of DMSO (*m*) on logarithmic solubility of PPD at *T* = 298.2 K to 318.2 K was also studied and results are presented in Figure 2. The results suggested linear increase in the logarithm solubility of PPD with increase in mass fraction of DMSO in binary DMSO + water systems at each temperature point studied. The solubility of PPD was found to increase significantly from neat water to neat DMSO. Hence, DMSO could be used as a potential cosolvent in solubility enhancement of PPD in an aqueous media such as water.

### 2.2. Ideal Solubilities and Activity Coefficients for Solute-Solvent Molecular Interactions

The ideal solubility (*x*^idl^) values for PPD were calculated using Equation (1) and results are tabulated in Table 1. The ideal solubilities of PPD were recorded in the range of 5.50 × 10^−2^ to 8.22 × 10^−2^ within the temperature range of *T* = 298.2 to 318.2 K. The ideal solubilities of PPD were significantly higher than its mole fraction solubilities in neat water. However, these values were lower than mole fraction solubilities of PPD in neat DMSO at each temperature point studied. Because of significant solubility of PPD in DMSO, it can also be used as an ideal cosolvent for solubility enhancement of PPD.

The values of activity coefficient (*γ*_i_) for PPD in binary DMSO + water systems at *T* = 298.2 K to 318.2 K were calculated using Equation (2) and results are tabulated in Table 2. The activity coefficient for PPD was found larger in neat water at each temperature point. However, the activity coefficient for PPD was lowest in neat DMSO at each temperature point. The activity coefficients for PPD were found to be decreasing significantly from neat water to neat DMSO. The larger activity coefficients for PPD in neat water were possible due to the lowest solubility of PPD in water. Overall, these results suggested maximum solute-solvent interactions in PPD-DMSO in comparison with PPD-water.

### 2.3. Thermodynamic Behavior of PPD

The values of different thermodynamic parameters for PPD estimated using van’t Hoff and Gibbs equations (Equations (3)–(6)) in binary DMSO + water systems and neat solvents are tabulated in Table 3.

The apparent standard enthalpy (Δ_sol_*H*^0^) values for PPD dissolution in binary DMSO + water systems and neat solvents were recorded as positive values (6.10 to 31.35 kJ mol^−1^), suggesting endothermic dissolution of PPD in all binary solvent systems and neat solvents [21,22].

The Δ_sol_*H*^0^ values for PPD dissolution were found to be decreasing with increase in the mass fraction of DMSO in binary DMSO + water systems and solubility values of PPD. Therefore, the highest Δ_sol_*H*^0^ value was recorded in neat water (31.35 kJ mol^−1^; *m* = 0.0), whereas, the lowest value was found in neat DMSO (6.10 kJ mol^−1^; *m* = 1.0). The apparent standard Gibbs free energy (Δ_sol_*G*^0^) values for PPD dissolution in binary DMSO + water systems were also found as positive values (2.14 to 29.89 kJ mol^−1^) as shown in Table 3. The Δ_sol_*G*^0^ values for PPD dissolution were also found to be decreasing with increase in the mass fraction of DMSO in binary DMSO + water systems and solubility values of PPD. The highest and lowest Δ_sol_*G*^0^ values for PPD dissolution were found in neat water (29.89 kJ mol^−1^; *m* = 0.0) and neat DMSO (2.14 kJ mol^−1^; *m* = 1.0), respectively.

The apparent standard entropy (Δ_sol_*S*^0^) values for PPD dissolution in binary DMSO + water systems were also recorded as positive values (4.74 to 13.01 J mol^−1^ K^−1^), suggesting entropy-driven dissolution of PPD in all DMSO + water systems and neat solvents [22]. The average Δ_sol_*H*^0^, Δ_sol_*G*^0^ and Δ_sol_*S*^0^ values for PPD were computed as 18.71 kJ mol^−1^, 15.99 kJ mol^−1^ and 8.83 J mol^−1^ K^−1^ with relative uncertainties of 0.44, 0.57 and 0.32, respectively. Overall, the dissolution process of PPD was found to be endothermic and entropy-driven in all cosolvent mixtures and neat solvents studied [21,22].

### 2.4. Enthalpy–Entropy Compensation Analysis for Solvation Property of PPD

**Figure 3:**
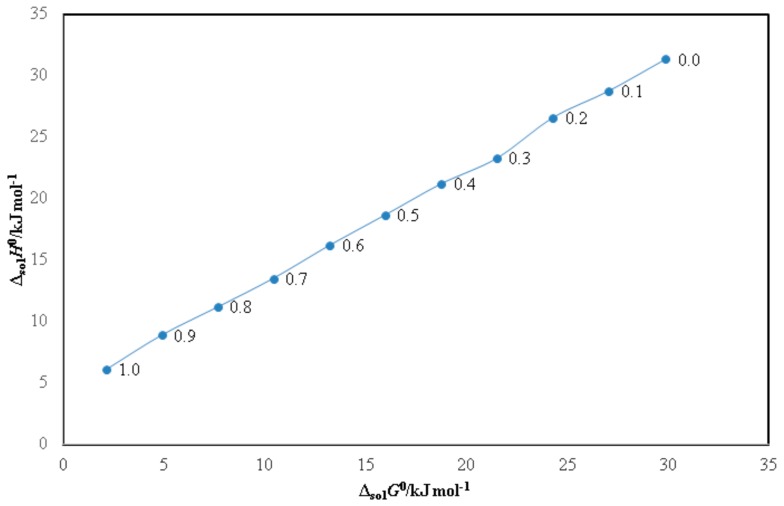
Δ_sol_*H*^0^ vs. Δ_sol_*G*^0^ enthalpy–entropy compensation plot for solubility of PPD in binary DMSO + water mixtures at *T*_hm_ of 308 K.

The results of enthalpy–entropy compensation analysis for PPD in binary DMSO + water systems and neat solvents are shown in Figure 3. It was observed that PPD in all binary DMSO + water systems and neat solvents showed linear Δ_sol_*H*° vs. Δ_sol_*G*° plot with a positive slope value of > 1.0 with *R*^2^ value of > 0.99. Based on these results, the driving mechanism for PPD solvation is as an enthalpy-driven in all binary DMSO + water systems and neat solvents. It was possible due to higher solvation of PPD in neat DMSO molecules in comparison with its solvation behavior in neat water molecules [12]. This solvation behavior of PPD in binary DMSO + water systems was in accordance with those reported for solvation properties of various weakly soluble drugs such as 6-methyl-2-thiouracil, sinapic acid, naringin and bergenin in binary DMSO + water mixtures [12,14,15,16].

### 2.5. Computation Validation

The correlation between experimental and model solubility of PPD was performed using root mean square deviations (*RMSD*) and correlation coefficient (*R*^2^). The results of the van’t Hoff model correlation (Equation (7)) for PPD in binary DMSO + water systems and neat solvents are tabulated in Table 4. The values of *RMSD* for PPD in binary DMSO + water systems and neat solvents were found as (0.27 to 2.18)%. The overall *RMSD* for this correlation was obtained as 1.30%. *RMSD* is deviation between experimental and model/theoretical solubility and it had no correlation with mass fraction of the cosolvent. Its decrease or increase with mass fraction had no significance [12,13]. The average relative uncertainties in model parameters *a* and *b* were obtained as 0.33 and 0.44, respectively. In general, the value of model parameter *a* was found to be increasing slightly with increase in the mass fraction of DMSO in DMSO + water systems. This enhancement was recorded for up to *m* = 0.9. After *m* = 0.9, there was little decrease in the value of model parameter *a*. This change (increase or decrease) in model parameter *a* was not significant. However, the value of model parameter *b* was found to be increasing significantly with increase in the mass fraction of DMSO in DMSO + water systems. This enhancement was recorded for up to *m* = 1.0. The values of *R*^2^ for van’t Hoff correlation were computed as 0.9930 to 0.9990. The data of *RMSD* (lower values) and *R*^2^ (higher values) recorded for the van’t Hoff model suggested good correlation of experimental solubility data of PPD with the van’t Hoff model.

The results of the Apelblat model correlation (Equation (8)) for PPD in binary DMSO + water systems are tabulated in Table 5. The values of *RMSD* for PPD in binary DMSO + water systems and neat solvents were computed as (0.20 to 1.28)%. The overall *RMSD* for Apelblat correlation was computed as 0.79%. The average relative uncertainties in model parameters *A, B*, and *C* were recorded as 0.61, 0.63 and 0.60, respectively. It was observed that the values of model parameter *A* and C were found to be increasing significantly with increase in the mass fraction of DMSO in DMSO + water systems. This enhancement was recorded for up to *m* = 1.0. However, the value of model parameter *B* was found to be decreasing with increase in the mass fraction of DMSO in DMSO + water systems. This enhancement was recorded for up to *m* = 0.9. The *R*^2^ values for this correlation were computed as 0.9984 to 0.9999. The data of *RMSD* (lower values) and *R*^2^ (higher values) recorded for the Apelblat model again suggested good correlation of experimental solubility data of PPD with the Apelblat model.

**Figure 4:**
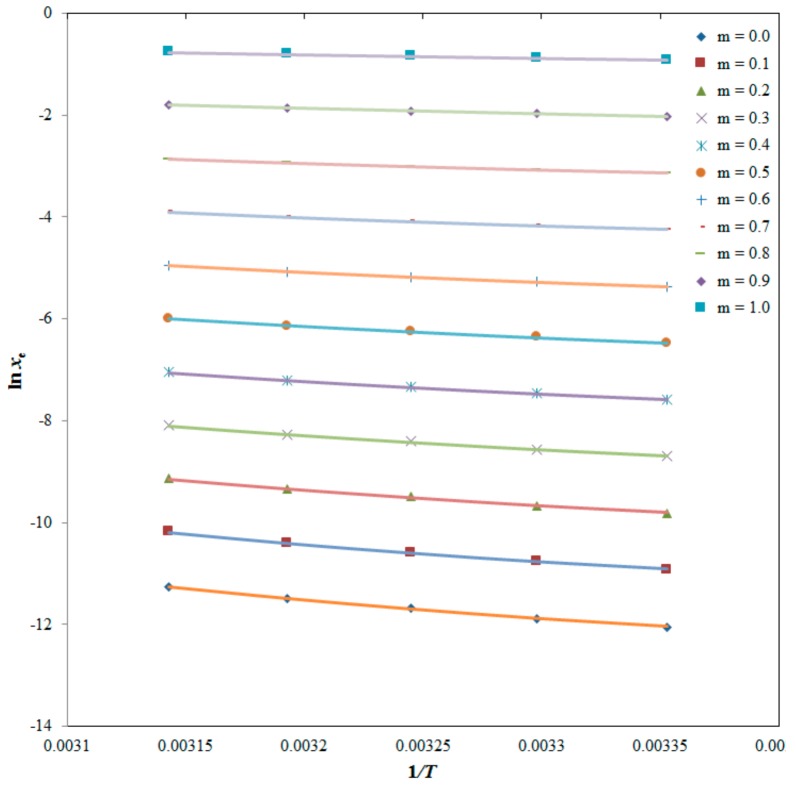
Correlation of ln *x*_e_ values of PPD with the Apelblat model in binary DMSO + water mixtures at *T* = 298.2 K to 318.2 K (Apelblat solubilities are represented by solid lines and experimental solubilities of PPD are represented by symbols).

The curve fitting between experimental and Apelblat solubilities of PPD are shown in Figure 4, suggesting good correlation of experimental solubilities of PPD with the Apelblat model.

The results of the Yalkowsky–Roseman model correlation (Equation (9)) for PPD in binary DMSO + water systems and neat solvents are tabulated in Table 6. The values of *RMSD* for the Yalkowsky–Roseman model correlation were computed as (0.61 to 2.25)%. The overall *RMSD* for this correlation was computed as 1.33%. The data of *RMSD* (lower values) recorded for the Yalkowsky model again suggested good correlation of experimental solubility data of PPD with the Yalkowsky model.

The results of the Jouyban–Acree (Equation (10)) and the Jouyban–Acree–van’t Hoff model (Equation (11)) correlation for PPD in binary DMSO + water system are listed in Table 7.

The overall *RMSD* value for the Jouyban–Acree model was computed as 0.74%. However, the overall *RMSD* value for the Jouyban–Acree–van’t Hoff model was computed as 0.62%. Overall, all five theoretical models performed well as the value of overall *RMSD* was <2.0% for all models. Nevertheless, the Jouyban–Acree model has been considered to be the best model for this correlation as this model uses the fewest model parameters.

## 3. Materials and Methods

### 3.1. Materials

The molecule PPD with mass fraction purity of 0.972 was synthesized, recrystallized, characterized, and identified in the Laboratory of Pharmaceutical Chemistry, Northern Border University, Rafha, Saudi Arabia [17]. DMSO with mass fraction purity of 0.993 was procured from Fluka Chemica (Buchs, Switzerland). Chromatography grades methanol with mass fraction purity of 0.999 and acetic acid with mass fraction purity of 0.997 were procured from Sigma Aldrich (St. Louis, MO, USA). The water was obtained from Milli-Q water purification unit.

### 3.2. Evaluation of PPD Solubility in Various DMSO + Water Systems

In this work, an isothermal method was applied to achieve solid-liquid equilibrium and solubility determination of PPD in binary solvent system of DMSO + water [23]. The measurements were carried out within the temperature range of *T* = 298.2 K to 318.2 K at constant air pressure *p* = 0.1 MPa. The excess amount of PPD was dispersed in glass vial containing 1.0 g of binary solvent system (*m* = 0.1 to 0.9) or neat solvent (*m* = 0.0 or 1.0). Each experiment was performed at least for three times. The resultant mixtures were located in the WiseBath^®^ WSB Shaking Water Bath (Model WSB-18/30/-45, Daihan Scientific Co. Ltd., Seoul, Korea) for a definite temperature (uncertainty of 0.12 K) and allowed to equilibrate for 72 h [17,21]. At the end of 72 h i.e., equilibrium time, the samples were taken out from the shaker and allowed to settle PPD particles for 24 h [16,22]. The supernatants from each saturated solution were withdrawn carefully, diluted and analyzed for PPD concentration by reported high performance liquid chromatography (HPLC) technique at the wavelength for maximum absorbance (λ_max_) of 254 nm [17]. The binary solvent system of methanol and acetic acid (99:1% *v*/*v*) was used as mobile phase for HPLC analysis of PPD. The *x*_e_ values of PPD were obtained using its standard equations reported in the literature [21,22].

### 3.3. Ideal Solubilities and Activity Coefficients for Solute-Solvent Molecular Interactions

The *x*^idl^ value of PPD was computed using the following equation [24]:

(1) $$\ln\;x^{\mathrm{idl}}=\;\frac{-\Delta H_{\mathrm{fus}}\left(T_{\mathrm{fus}}-T\right)}{RT_{\mathrm{fus}}T}+\left(\frac{\Delta C_{p}}{R}\right)[\frac{T_{\mathrm{fus}}-T}{T}+\ln\left(\frac{T}{T_{\mathrm{fus}}}\right)]\;$$

where *T* = absolute temperature; *T*_fus_ = fusion/melting temperature of PPD; *R* = universal gas constant; ∆*H*_fus_ = molar fusion enthalpy of PPD and ∆*C*_p_ = difference in the molar heat capacity of solid form with that of liquid form [24,25]. The values of *T*_fus_, ∆*H*_fus_ and ∆*C*_p_ for PPD were obtained as 476.43 K, 24.51 kJ mol^−1^ and 51.44 J mol^−1^ K^−1^, respectively from reference [17].

Now using Equation (1), the *x*^idl^ values for PPD were computed.

The values of *γ*_i_ for PPD in various DMSO + water systems were computed using the following equation [24,26]:

(2) $$\gamma_{i}=\;\frac{x^{\mathrm{idl}}}{x_{e}}$$

In which *x*_e_ ≠ 0. Using activity coefficients, the molecular interactions were evaluated.

### 3.4. Thermodynamic Behavior of PPD

Dissolution thermodynamics of PPD in various solvent mixtures of DMSO + water was studied by estimating apparent thermodynamic analysis based on van’t Hoff and Gibbs equations. The van’t Hoff equation was applied to estimate thermodynamic properties of PPD in investigated binary solvent systems is obtained from the following equation at mean harmonic temperature (*T*_hm_) which was computed as 308 K within the temperature range of *T* = 298.2 to 318.2 K [24,27]:

(3) ∂ln xe∂1T−1ThmP= −ΔsolH0R

where *x*_e_ = mole fraction solubility of PPD in binary solvent system of DMSO + water; *T* = absolute temperature and *R* = universal gas constant. Here, *T* ≠ 0 and *T*_hm_ ≠ 0. By plotting ln *x*_e_ versus 1T−1Thm, the values of Δ_sol_*H*^0^ and Δ_sol_*G*^0^ for dissolution of PPD were calculated from the slope and intercept, respectively by applying the following equations [28]:

(4) ΔsolH0=−R∂ln xe∂1T−1ThmP

(5) $$\;\Delta_{\mathrm{sol}}G^{0}=\;-RT_{\mathrm{hm}}\times \mathrm{intercept}\;$$

The intercept values for PPD in binary DMSO + water systems were obtained from van’t Hoff graphs plotted between ln *x*_e_ and 1T−1Thm.  Finally, the Δ_sol_*S*^0^ values for PPD dissolution in binary DMSO + water systems were computed using Gibbs equation given below [24,27,28]:

(6) $$\;\;\Delta_{\mathrm{sol}}S^{0}\;\frac{\Delta_{\mathrm{sol}}H^{0}-\Delta_{\mathrm{sol}}G^{0}}{T_{\mathrm{hm}}}\;\;$$

### 3.5. Enthalpy–Entropy Compensation Analysis

The solvation properties of PPD in binary DMSO + water systems were evaluated using an enthalpy–entropy compensation analysis [27,29]. Such analysis was performed by plotting the weighted graphs of Δ_sol_*H*° vs. Δ_sol_*G*° at *T*_hm_ value of 308 K [29].

### 3.6. Computational Validation

The *x*_e_ values of PPD were fitted using five different computational models including the van’t Hoff, Apelblat, Yalkowsky–Roseman, Jouyban–Acree and Jouyban–Acree–van’t Hoff models [29,30,31,32,33].

The van’t Hoff model solubility (*x*^van’t^) of PPD in binary DMSO + water mixtures and neat solvents was computed using the following equation [29]:

(7) $$\ln\;x^{\mathrm{van}\prime t}=a+\frac{b}{T}$$

where *a* and *b* = model parameters of Equation (7) which were estimated by constructing plots between ln *x_e_* values of PPD and of 1/*T*.

The Apelblat model solubility (*x*^Apl^) of PPD in binary DMSO + water systems and neat solvents was computed using the following equation [30,31]:

(8) $$\ln\;x^{\mathrm{Apl}}=A+\frac{B}{T}+\;C\ln\left(T\right)$$

where *A, B*, and C = model parameters Equation (8) which were estimated by nonlinear multivariate regression analysis of *x*_e_ values of PPD tabulated in Table 1 [29].

The logarithmic solubility of the Yalkowsky model (log *x*^Yal^) for PPD in binary DMSO + water systems and neat solvents was computed using the following equation [32]:

(9) $$\;\mathrm{Log}x^{\mathrm{Yal}}=m_{1}logx_{1}+\;m_{2}logx_{2}$$

where *x*_1_ = mole fraction solubility of PPD in neat DMSO; *x*_2_ = mole fraction solubility of PPD in neat water; *m*_1_ = mass fraction of neat DMSO and *m*_2_ = mass fraction of neat water in the absence of solute.

The Jouyban–Acree model solubility (*x*_m,T_) of PPD in binary DMSO + water systems was computed by applying the following Equation [33]:

(10) $$\ln x_{m,T}=m_{1}\ln x_{1}+m_{2}\;\ln\;x_{2}+\;\left\lceil m_{1},m_{2},\overset{2}{\underset{i=0}{\sum }},\frac{J_{i}}{T},{\left(m_{1}-m_{2}\right)}^{i}\right\rceil$$

where *J*_i_ = model parameter of Equation (10) and it was estimated from no-intercept regression analysis [34].

The Jouyban–Acree–van’t Hoff solubility of PPD in binary DMSO + water systems was computed using the following equation [35]:

(11) $$\ln\;x_{m,T}=m_{1}\left(A_{1}+\;\frac{B_{1}}{T}\right)+m_{2}\;\left(A_{2}+\;\frac{B_{2}}{T}\right)+\;\left[\frac{m_{1}m_{2}}{T}\;\overset{2}{\underset{i=0}{\sum }}J_{i}{\left(m_{1}-m_{2}\right)}^{i}\right]$$

where *A_1_*, *B_1_*, *A_2_*, *B_2_* and *J_i_* = model parameters of Equation (11).

## 4. Conclusions

The solubility, solution thermodynamics and solvation behavior of PPD in binary DMSO + water systems were studied at *T* = 298.2 K to 318.2 K and *p* = 0.1 MPa using experimental and various computational approaches. The solubilities of PPD were found to be increasing with raise in temperature and increase in the mass fraction of DMSO in binary DMSO + water systems in all cases. The highest and lowest solubilities of PPD were found in neat DMSO and neat water, respectively. The experimental solubilities of PPD were correlated well by van’t Hoff, Apelblat, Yalkowsky–Roseman, Jouyban–Acree and Jouyban–Acree–van’t Hoff models with overall *RMSD* of <2.0% in all DMSO + water systems. The results of activity coefficients showed maximum molecular interaction in PPD-DMSO. The dissolution of PPD was observed as endothermic and entropy-driven in all binary DMSO + water systems and neat solvents. Enthalpy–entropy compensation analysis indicated enthalpy-driven mechanism as the driven mechanism for solvation property of PPD.

## Figures and Tables

**Table 1:** The *x*_e_ values of PPD against mass fraction value of DMSO (*m*) in binary DMSO + water mixtures at *T* = 298.2 K to 318.2 K and *p* = 0.1 MPa ^a^.

m	x_e_				
	T = 298.2 K	T = 303.2 K	T = 308.2 K	T = 313.2 K	T = 318.2 K
0.0	5.82 × 10^−6^	6.94 × 10^−6^	8.42 × 10^−6^	1.02 × 10^−5^	1.30 × 10^−5^
0.1	1.81 × 10^−5^	2.13 × 10^−5^	2.53 × 10^−5^	3.03 × 10^−5^	3.79 × 10^−5^
0.2	5.47 × 10^−5^	6.31 × 10^−5^	7.54 × 10^−5^	8.78 × 10^−5^	1.08 × 10^−4^
0.3	1.69 × 10^−4^	1.91 × 10^−4^	2.26 × 10^−4^	2.56 × 10^−4^	3.05 × 10^−4^
0.4	5.05 × 10^−4^	5.71 × 10^−4^	6.50 × 10^−4^	7.40 × 10^−4^	8.68 × 10^−4^
0.5	1.54 × 10^−3^	1.73 × 10^−3^	1.93 × 10^−3^	2.17 × 10^−3^	2.49 × 10^−3^
0.6	4.68 × 10^−3^	5.14 × 10^−3^	5.68 × 10^−3^	6.27 × 10^−3^	7.08 × 10^−3^
0.7	1.48 × 10^−2^	1.56 × 10^−2^	1.69 × 10^−2^	1.84 × 10^−2^	2.04 × 10^−2^
0.8	4.34 × 10^−2^	4.62 × 10^−2^	4.96 × 10^−2^	5.33 × 10^−2^	5.76 × 10^−2^
0.9	1.32 × 10^−1^	1.40 × 10^−1^	1.47 × 10^−1^	1.56 × 10^−1^	1.65 × 10^−1^
1.0	4.00 × 10^−1^	4.16 × 10^−1^	4.32 × 10^−1^	4.49 × 10^−1^	4.67 × 10^−1^
x^idl^	5.50 × 10^−2^	6.10 × 10^−2^	6.75 × 10^−2^	7.45 × 10^−2^	8.22 × 10^−2^

^a^ The standard uncertainties *u* are *u*(*T*) = 0.12 K, *u*_r_(*m*) = 0.1%, *u*(*p*) = 0.003 MPa and *u*_r_(*x*_e_) = 1.38%.

**Table 2:** The estimated values of *γ*_i_ for PPD in binary DMSO + water mixtures (*m*) at *T* = 298.2 K to 318.2 K.

m	γ_i_				
	T = 298.2 K	T = 303.2 K	T = 308.2 K	T = 313.2 K	T = 318.2 K
0.0	9460.000	8800.000	8020.000	7340.000	6340.000
0.1	3036.053	2863.900	2671.535	2464.940	2173.383
0.2	1007.450	967.752	895.279	849.738	764.882
0.3	325.566	319.655	298.478	291.063	269.649
0.4	109.123	106.877	103.965	100.798	94.754
0.5	35.637	35.245	34.901	34.347	32.997
0.6	11.761	11.865	11.885	11.884	11.617
0.7	3.802	3.905	3.985	4.038	4.026
0.8	1.266	1.320	1.359	1.398	1.425
0.9	0.417	0.435	0.457	0.477	0.496
1.0	0.137	0.146	0.156	0.165	0.175

**Table 3:** Apparent thermodynamic quantities (Δ_sol_*H*^0^, Δ_sol_*G*^0^ and Δ_sol_*S*^0^) and *R*^2^ values for PPD dissolution in binary DMSO + water mixtures ^b^.

Parameters	*m* = 0.0	*m* = 0.1	*m* = 0.2	*m* = 0.3	*m* = 0.4	*m* = 0.5	*m* = 0.6	*m* = 0.7	*m* = 0.8	*m* = 0.9	*m* = 1.0
Δ_sol_*H*^0^/kJ mol^−1^	31.35	28.75	26.59	23.28	21.22	18.68	16.21	13.51	11.20	8.90	6.10
Δ_sol_*G*^0^/kJ mol^−1^	29.89	27.06	24.30	21.51	18.77	15.98	13.22	10.42	7.68	4.89	2.14
Δ_sol_*S*^0^/J mol^−1^ K^−1^	4.74	5.49	7.44	5.73	7.97	8.78	9.69	10.02	11.43	13.01	12.85
*R* ^2^	0.9941	0.9931	0.9947	0.9949	0.9954	0.9964	0.9958	0.9946	0.9963	0.9990	0.9991

^b^ The average uncertainties are *u*(Δ_sol_*H*^0^) = 0.44 kJ mol^−1^, *u*(Δ_sol_*G*^0^) = 0.57 kJ mol^−1^ and *u*(Δ_sol_*S*^0^) = 0.32 J mol^−1^ K^−1^.

**Table 4:** The van’t Hoff model parameters (*a* and *b*), *R*^2^ and *RMSD* values for PPD in binary DMSO + water mixtures ^c^.

m	a	b	R^2^	RMSD (%)	Overall RMSD (%)
0.0	0.55	−3765.70	0.9939	2.18	
0.1	0.64	−3454.00	0.9930	2.17	
0.2	0.87	−3194.30	0.9946	1.92	
0.3	0.67	−2796.20	0.9947	1.62	
0.4	0.94	−2549.80	0.9953	1.42	
0.5	1.04	−2244.60	0.9963	1.15	
0.6	1.15	−1947.30	0.9957	1.14	
0.7	1.19	−1623.00	0.9944	1.20	
0.8	1.36	−1345.40	0.9962	1.02	
0.9	1.56	−1069.60	0.9989	0.27	
1.0	1.54	−733.05	0.9990	0.28	1.30

^c^ The average relative uncertainties are *u*(*a*) = 0.33 and *u*(*b*) = 0.44.

**Table 5:** Apelblat model parameters (*A, B*, and C), *R*^2^ and *RMSD* for PPD in binary DMSO + water mixtures ^d^.

m	A	B	C	R^2^	RMSD (%)	Overall RMSD (%)
0.0	−750.34	30686.31	111.51	0.9996	1.17	
0.1	−740.88	30569.14	110.12	0.9996	1.07	
0.2	−587.97	23822.39	87.45	0.9994	1.19	
0.3	−451.37	17943.26	67.13	0.9984	1.28	
0.4	−441.66	17756.69	65.73	0.9997	0.82	
0.5	−331.65	13018.47	49.41	0.9995	0.62	
0.6	−324.19	12979.37	48.31	0.9997	0.67	
0.7	−311.35	12717.06	46.41	0.9998	0.65	
0.8	−214.94	8578.56	32.12	0.9999	0.63	
0.9	−61.67	1829.38	9.39	0.9995	0.20	
1.0	−58.21	2007.18	8.87	0.9999	0.41	0.79

^d^ The average relative uncertainties are *u*(*A*) = 0.61, *u*(*B*) = 0.63 and *u*(*C*) = 0.60.

**Table 6:** Log *x*^Yal^ values of PPD calculated by Yalkowsky model in binary DMSO + water mixtures at *T* = 298.2 K to 318.2 K.

m	Log x^Yal^					*RMSD* (%)	Overall *RMSD* (%)
	298.15	303.15	308.15	313.15	318.15		
0.1	−4.75	−4.68	−4.60	−4.52	−4.43	1.95	
0.2	−4.26	−4.20	−4.13	−4.06	−3.97	1.49	
0.3	−3.78	−3.72	−3.66	−3.59	−3.51	2.25	
0.4	−3.29	−3.24	−3.19	−3.13	−3.06	0.72	
0.5	−2.81	−2.76	−2.71	−2.66	−2.60	1.43	
0.6	−2.33	−2.29	−2.24	−2.20	−2.15	0.68	
0.7	−1.84	−1.81	−1.77	−1.74	−1.69	1.78	
0.8	−1.36	−1.33	−1.30	−1.27	−1.24	0.61	
0.9	−0.88	−0.85	−0.83	−0.81	−0.78	1.10	1.33

**Table 7:** The parameters of Jouyban–Acree and Jouyban–Acree–van’t Hoff models for PPD in binary DMSO + water systems.

System	Jouyban–Acree	Jouyban–Acree–van’t Hoff
		*A*_1_ 1.54 *B*_1_ −733.05 *A*_2_ 0.55 *B*_2_ −3765.7 *J*_i_ 25.32 0.62
DMSO + water	*J*_i_ 28.19	
	
	
*RMSD* (%)	0.74	

## Nomenclature

*T*	Absolute temperature (K)
*P*	Air pressure (MPa)
*M*	Mass fraction of DMSO in DMSO + water mixtures
*m*_1_	Mass fraction of neat DMSO
*m*_2_	Mass fraction of neat water
*x*_e_	Experimental mole fraction solubility of PPD
*x* ^idl^	Ideal solubility of PPD in mole fraction
*x* ^van’t^	van’t Hoff model solubility of PPD in mole fraction
*x* ^Apl^	Apelblat model solubility of PPD in mole fraction
*x* ^Yal^	Yalkowsky model solubility of PPD in mole fraction
*x*_m,T_	Jouyban–Acree model solubility of PPD in mole fraction
*x*_1_	Mole fraction solubility of PPD in neat DMSO
*x*_2_	Mole fraction solubility of PPD in neat water
*γ*_i_	Activity coefficient of PPD
*R* ^2^	Correlation coefficient
*RMSD*	Root mean square deviations (%)
*a* and *b*	Parameters of van’t Hoff model
*A*, *B*, and *C*	Parameters of Apelblat model
*J*_i_	Parameter of Jouyban–Acree model
*A*_1_, *B*_1_, *A*_2_, and *B2*	Parameter of Jouyban–Acree–van’t Hoff model
Δ_sol_*H*^0^	Apparent standard dissolution enthalpy (kJ mol^−1^)
Δ_sol_*G*^0^	Apparent standard Gibbs free energy (kJ mol^−1^)
Δ_sol_*S*^0^	Apparent standard dissolution entropy (J mol^−1^ K^−1^)
*T*_hm_	Mean harmonic temperature (K)
*T*_fus_	Fusion temperature (K)
*λ*_max_	Wavelength for maximum absorbance (nm)
*R*	Universal gas constant (J mol^−1^ K^−1^)
Δ*H*_fus_	Molar fusion enthalpy (kJ mol^−1^)
Δ*C*_p_	Difference in molar heat capacity (J mol^−1^ K^−1^)
